# Molecular detection of trypanosomes of the *Trypanosoma livingstonei* species group in diverse bat species in Central Cameroon

**DOI:** 10.1007/s00436-024-08303-0

**Published:** 2024-07-22

**Authors:** K. J. A. Tsague, E. M. Bakwo Fils, J. P. Atagana, D. W. Mbeng, L. Palm, T. Tchuinkam, J. Schaer

**Affiliations:** 1 Laboratory of Biological Sciences, Faculty of Sciences of University of Maroua, Maroua, Cameroon; 2 Vector Borne Diseases Laboratory of the Research Unit for Biology and Applied Ecology (VBID-RUBAE), University of Dschang, Dschang, Cameroon; 3 Department of Environmental Sciences, Higher Institute of Agriculture, Forestry, Water and Environment (HIAFWE), University of Ebolowa, Ebolowa, Cameroon; 4 Department of Biological Science, Faculty of Science, University of Ngaoundere, Ngaoundere, Cameroon; 5 Department of Molecular Parasitology, Institute of Biology, Humboldt University, Berlin, Germany; 6 Department of Biology, Muni University, Arua, Uganda; 7 Museum Für Naturkunde, Leibniz-Institute for Evolution and Biodiversity Science, Berlin, Germany; 8 Department of Biological Sciences, Macquarie University, Sydney, Australia

**Keywords:** *Trypanosoma*, Bats, *T. cruzi*, Co-infection, Africa

## Abstract

Bats are hosts for diverse *Trypanosoma* species, including trypanosomes of the *Trypanosoma cruzi* clade. This clade is believed to have originated in Africa and diversified in many lineages worldwide. In several geographical areas, including Cameroon, no data about trypanosomes of bats has been collected yet. In this study, we investigated the diversity and phylogenetic relationships of trypanosomes of different bat species in the central region of Cameroon. Trypanosome infections were detected in six bat species of four bat families, namely Hipposideridae, Pteropodidae, Rhinolophidae, and Vespertilionidae, with an overall prevalence of 29% and the highest infection rate in hipposiderid bat species*.* All trypanosomes were identified as belonging to the *Trypanosoma livingstonei* species group with one clade that might represent an additional subspecies of *T. livingstonei*. Understanding the prevalence, distribution, and host range of parasites of this group contributes to our overall knowledge of the diversity and host specificity of trypanosome species that phylogenetically group at the base of the *T. cruzi* clade.

**Supplementary Information:**

The online version contains supplementary material available at 10.1007/s00436-024-08303-0.

## Introduction

Bats are hosts to a diversity of eukaryotic protozoan parasites, including trypanosomes, *Babesia*, haemosporidians, and *Leishmania* (e.g., Gardner and Molyneux 1987; Lima et al. 2013; Schaer et al. 2013; de Souza et al. 2023). Trypanosomes (genus *Trypanosoma*) are flagellated kinetoplastid blood parasites that are transmitted by leeches and various bloodsucking arthropods and have adapted to infect various classes of vertebrates that comprise several mammalian groups and include species that are a threat to human and animal health (Simpson et al. 2006; Morrison et al. 2016; Büscher et al. 2017). Bats are recognized as hosts for diverse *Trypanosoma* species, and studies have revealed that the majority of identified bat trypanosomes fall within the *Trypanosoma cruzi* clade with evidence that bats played an important role in the evolution of the *T. cruzi* species group (e.g., Austen and Barbosa 2021). However, the knowledge about the diversity of bat trypanosomes, their vectors, distribution, and the evolutionary history of trypanosomes is still limited (e.g., Hamilton et al. 2012; Lima et al. 2013; Clement et al. 2020). In several geographical areas, including Cameroon, no data about trypanosomes of bats has been collected yet. With 112 species of bats, Cameroon is one of the hot spots of bat diversity in Africa (ACR 2022). In the current study, we investigate the diversity and phylogenetic relationships of trypanosomes of different bat species in the central region of Cameroon using molecular methods.

## Material and methods

Sampling of bats was conducted in the central region of Cameroon in the dry and wet season between February 2016 and December 2019 across different habitat types like forest, savanna, and cultured farmland as described in Tsague et al. (2022). Bat individuals were captured using ground-level mist nets, and different identification keys were used for morphological species identification (e.g., Rosevear 1965; Patterson and Webala 2012). Small blood samples were collected by venipuncture of the uropatagial vein. A thin blood smear and blood dots on Whatman filter paper (GE Healthcare) were collected from every individual, before it got released at the capture site. The blood smears were dried and fixed in 99–100% (vol/vol) methanol solution for 3 s and subsequently stained with 10% Giemsa solution for 20 min and air-dried. Blood smears were screened for the presence of trypanosome parasites with light microscopy (Leica DMLB 1000) at a magnification of × 400 and × 1000.

The QIAGEN DNeasy blood and tissue extraction kit (Hilden, Germany) was used to extract whole genomic DNA from the dried blood dots following Schaer et al. (2013). PCRs were performed using the AllTaq Master Mix Kit (QIAGEN) with 4 µl DNA and 1 µl of each primer (10 mM). A nested-PCR approach was used for the amplification of about 600 bp of the trypanosome’s small subunit 18S ribosomal RNA gene (18S rRNA) following Noyes et al. (1999) using the primers TRY927F/R for the outer reaction and SSU561F/R for the nested reaction. PCR products were sequenced with the amplification primers and Sanger-sequenced. All nucleotide sequences were assessed for quality and manually edited in the software Geneious Prime 2023.1.2 (https://www.geneious.com) and amplification and sequencing were repeated for samples with low-quality sequences. Trypanosome nucleotide sequences were aligned with reference sequences, obtained from NCBI GenBank, using the MAFFT algorithm (Katoh et al. 2002). The GenBank accession numbers are provided in the respective phylogenetic tree figure. The sequence alignment for the analysis of *Trypanosoma* taxa comprised 94 sequences and a length of 667 nt. The software *modeltest-ng* 0.1.7 was used to test different DNA substitution models and the maximum likelihood (ML) analysis was carried out in raxmlGUI 2.0.10 (Darriba et al. 2020) using the model TIM3 + I + G with 10,000 bootstrap iterations and the taxon *Trypanosoma lewisi* as outgroup. The phylogenetic tree was displayed in FigTree v1.4.4 (http://tree.bio.ed.ac.uk/software/figtree/). Haplotype networks were constructed in PopART v.1.7 (Leigh and Bryant 2015) using the median-joining algorithm with default settings. The networks were labeled according to the bat host species from which the *Trypanosoma* sequence was amplified. Some of the (high-quality) sequences contained ambiguous base calls (with double nucleotide peaks) that point to mixed haplotype infections (Table S1) and were therefore excluded from haplotype network analyses (Table S1).

## Results and discussion

A total of 159 bats belonging to four bat families, nine genera, and 13 species were investigated and screened with PCR. *Trypanosoma* parasite DNA was detected in samples of 46 bat individuals, corresponding to an overall prevalence of 29% (Table 1). Infections were detected in bat species of all four investigated bat families Hipposideridae, Pteropodidae, Rhinolophidae, and Vespertilionidae.

**Table 1:** Investigated bat species and corresponding prevalence of trypanosome infections

Bat family	Bat species	Prevalence in % (# of infected/total individuals)
Hipposideridae	*Doryrhina cyclops*	70.0 (7/10)
	*Hipposideros abae*	0 (0/2)
	*Hipposideros curtus*	38.2 (13/34)
	*Hipposideros fuliginosus*	0 (0/19)
	*Hipposideros ruber*	76.0 (19/25)
Pteropodidae	*Eidolon helvum*	0 (0/1)
	*Epomops franqueti*	0 (0/15)
	*Epomophorus pusillus*	9.1 (3/33)
	*Rousettus aegyptiacus*	0 (0/1)
Rhinolophidae	*Rhinolophus alcyone*	0 (0/2)
	*Rhinolophus cf. landeri*	27.3 (3/11)
Vespertilionidae	*Glauconycteris humeralis*	0 (0/1)
	*Afronycteris nana*	20.0 (1/5)
Total		28.9 (46/159)

Highest prevalences of trypanosome infections were identified in three bat species of the bat family Hipposideridae, with 76% (19/25) infected individuals of *Hipposideros ruber*, 70% (7/10) of *Doryrhina cyclops*, and 38% (13/34) of *Hipposideros curtus.* No infections were recorded for *Hipposideros abae* (0/2) and *H. fuliginosus* (0/19). A low prevalence of trypanosome infections was documented from fruit bats (Pteropodidae), with 9% (3/33) infections in *Epomophorus pusillus* and none in *Eidolon helvum* (0/1), *Epomops franqueti* (0/15), and *Rousettus aegyptiacus* (0/1), though the sample size of the three latter species was quite low. Two species of *Rhinolophus* (Rhinolophidae) and two vespertilionid bat species were investigated in the study, with infections verified in one rhinolophid species, *Rhinolophus* cf. *landeri* (27%, 3/11) and in *Afronycteris nana* (20%, 1/5) (Vespertilionidae) (Table 1).

All infections were identified via PCR and sequencing. Comparison of the 18S rRNA trypanosome sequences of the 46 samples with reference sequences on NCBI GenBank (BLASTn) featured highest identity either with sequences of the species *Trypanosoma livingstonei*/*Trypanosoma* cf. *livingstonei* or a closely related *Trypanosoma* sp. taxon reported from African and European *Miniopterus* species (Clement et al. 2020; Szentivanyi et al. 2020). Our dataset comprised samples with trypanosome sequences of lower quality, but comparison of these sequences with reference sequences on NCBI GenBank (BLASTn) also featured highest identities with *T.* cf. *livingstonei*/*livingstonei*/sp. parasites (Table S1). High-quality trypanosome sequences from 28 bat samples were included in the subsequent haplotype network and phylogenetic analyses.

**Fig. 1:**
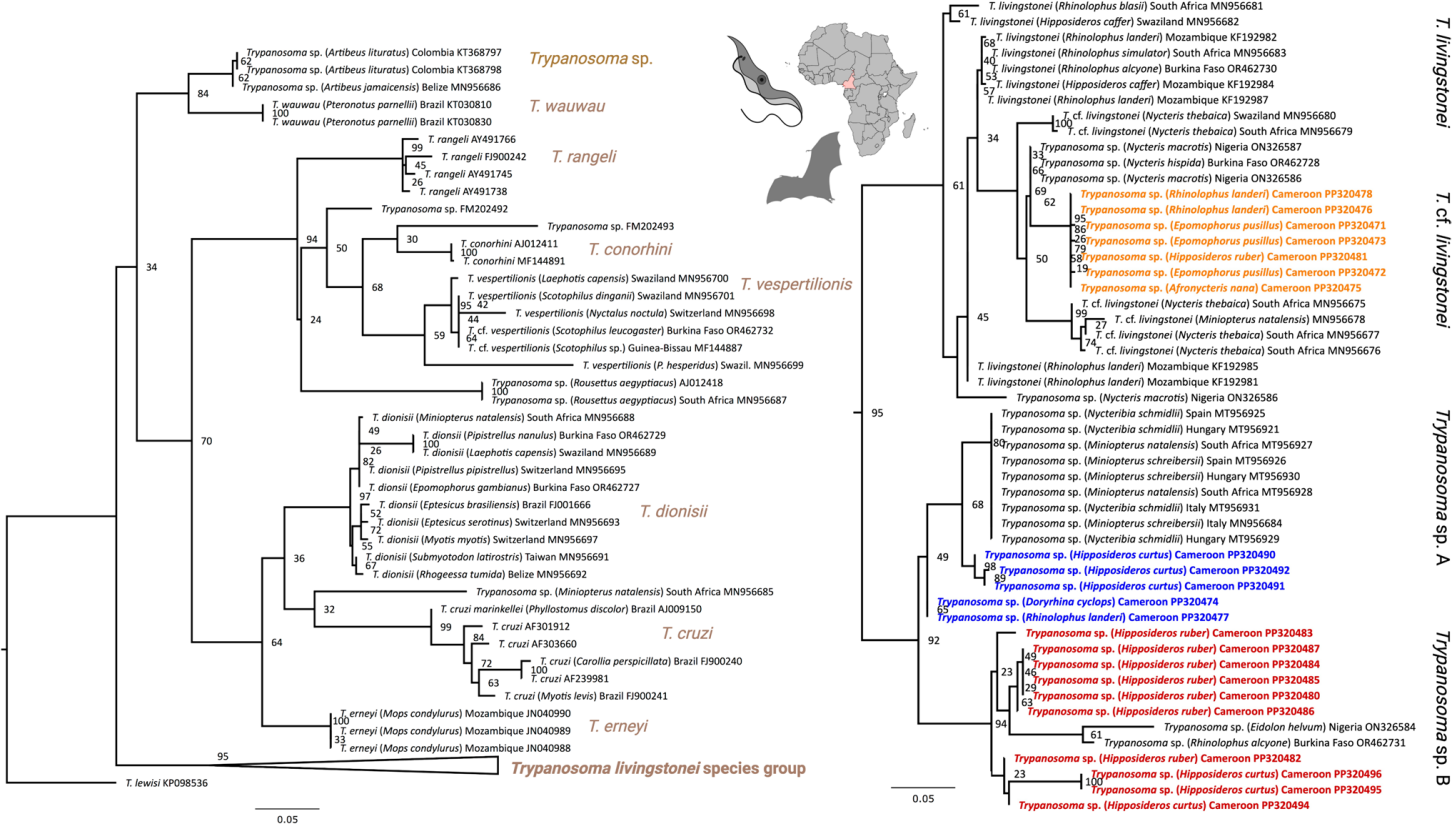
Maximum likelihood (ML) phylogeny of *Trypanosoma* parasites of the *Trypanosoma cruzi* clade. The 18S rRNA sequence alignment (length of 667 nt) for the analysis comprised 94 sequences. The ML analysis was carried out using the model TIM3 + I + G with 10,000 bootstrap iterations and the taxon *Trypanosoma lewisi* as outgroup. Bootstrap values (> 20) are given. All trypanosome sequences of the study (highlighted in bold) fall within the *Trypanosoma livingstonei* species group (collapsed on the left, uncollapsed on the right). Figure was assembled with BioRender.com

The 18S rRNA maximum likelihood phylogenetic analysis recovered the trypanosome sequences of the study within the wider *T. livingstonei* clade (with high support, bootstrap value of 95) (Fig. 1).

Within this *T. livingstonei* clade, the Cameroonian bat trypanosome sequences fall in three main subclades. The first main subclade that includes trypanosome sequences of *A. nana*, *E. pusillus*, *H. ruber*, and *R. landeri* from Cameroon (Fig. 1, highlighted in orange) falls within the group of *T*. cf. *livingstonei* parasites that are nested within *T. livingstonei* parasites. The trypanosome sequences of five *H. ruber*, *A. nana* (*n* = 1), and *R. landeri* (*n* = 1) represent one haplotype (haplotype H1), while each sequence of the three *E. pusillus* bats represents its own haplotype that differs from haplotype H1 by one base each (Supplementary Fig. S1A). Several branches within the *T. livingstonei/T*. cf. *livingstonei* are not well supported and a more complete taxon sampling and additional molecular markers are necessary to resolve the relationships among the wider *T. livingstonei* parasite clade (Fig. 1).

The second subclade includes the aforementioned *Trypanosoma* sp. clade from *Miniopterus* bat species (here termed *Trypanosoma* sp. A) and two sequences from *H. curtus* from Cameroon (Fig. 1, highlighted in blue) that group as sister group to *Trypanosoma* sp. A (with low support, bootstrap value = 68). Basal to this group, trypanosome sequences from *R. landeri* and *D. cyclops* (Fig. 1, highlighted in blue) from Cameroon form a separate clade albeit with low support (bootstrap value = 49). The sequences from *H. curtus* represent three different haplotypes, while the sequences of *R. landeri* and *D. cyclops* (*n* = 2) share one haplotype (Supplementary Fig. S1B). The third subclade, here termed *Trypanosoma* sp. B and supported with a high bootstrap value of 94, comprises two trypanosome parasites from Nigerian bat hosts plus the trypanosome sequences from *H. ruber* and the remaining sequences from *H. curtus* from Cameroon (Fig. 1, highlighted in red). Both main subclades, the wider *Trypanosoma* sp. A and B clades, group as sister clades with high support (bootstrap value 92). So, despite bat host specific clustering of some sequences (e.g., some trypanosome sequences of *H. ruber* and *H. curtus*), the results of shared haplotypes and phylogenetic clades of trypanosomes from different bat species point to an overall low host species specificity.

Unfortunately, no trypanosome parasite stages were detected in any of the blood smears of the infected bat samples which points to subpatent/low parasitemia trypanosome infections. In many wildlife hosts, trypanosome infections can be chronic and asymptomatic, leading to low levels of parasites in the bloodstream (e.g., Njiokou et al. 2006). Therefore, the research of wildlife trypanosomes often involves the use of hemoculture to culture trypanosomes from blood samples. This method helps in isolating and identifying different trypanosome species, facilitating, e.g., the microscopic study of the parasite morphology and provides large amounts of DNA for molecular and phylogenetic analyses (e.g., Lima et al. 2013). However, for our current study, we did not collect sufficient amounts of blood from each bat that would have been required for hemoculture. Thus, the findings of the study present a first snapshot of the diversity and prevalence of trypanosome taxa in bats in Central Cameroon, but further studies that include morphological characterization of the parasites and facilitate the analysis of additional phylogenetic markers are needed.

The trypanosome species *T. livingstonei* was originally described in bats from Mozambique (Lima et al. 2013). Since then, *T. livingstonei*, its putative subspecies *T*. cf. *livingstonei*, and the closely related *Trypanosoma* sp. A (Clement et al. 2020; Szentivanyi et al. 2020) have been reported from a diversity of African bat species, including the six different bat species in this study (e.g., Clement et al. 2020; Kamani et al. 2022; Thiombiano et al. 2023). The results recovered another subclade of *T. livingstonei*, the trypanosomes of the *Trypanosoma* sp. B group. Our data confirm and enlarge the diversity of the *T. livingstonei* species group, especially among trypanosomes of African bat species. Understanding the prevalence, distribution, and host range of parasites of the *T. livingstonei* parasite group contributes to our overall knowledge of the diversity and host specificity of trypanosomes species that originated from Africa and phylogenetically group at the base of the *T. cruzi* clade (Clement et al. 2020; Austen and Barbosa 2021). The species *T. cruzi* causes Chagas disease in humans and therefore identification and research of closely related trypanosome species is of importance (e.g., Beltz 2017). Understanding the diversity and phylogenetic relationships of bat trypanosomes is crucial for improving our knowledge of the broader group of parasites (Hamilton et al. 2012; Lima et al. 2012). Numerous trypanosome lineages within the *T. cruzi* clade may have originated in African bat species (e.g., Lima et al. 2013; Clement et al. 2020), highlighting the importance of targeted systematic sampling and molecular characterization of trypanosome species from African bats.

Of note, for the two bat species *D. cyclops* and *E. pusillus*, a high incidence of co-infections of trypanosomes and haemosporidian parasites was discovered. The haemosporidian infections in the samples of this study were identified in a previous study that used the same samples and, at that time, focused exclusively on infections with haemosporidian parasites (Tsague et al. 2022). Six out of the seven trypanosome-infected *D. cyclops* individuals featured infections with *Nycteria* parasites, while the three trypanosome-infected *E. labiatus* individuals were co-infected with *Hepatocystis* parasites (Tsague et al. 2022). To the best knowledge of the authors, this is the first time that co-infections with the two unrelated eukaryotic blood parasites, trypanosomes and haemosporidians (*Hepatocystis* or *Nycteria*), have been documented. The co-infections could be a result of a common transmission mechanism or a shared arthropod vector. However, the vectors for the trypanosomes of the study are unknown as are the vectors for *Nycteria* parasites (e.g., Schaer et al. 2015). Bat *Hepatocystis* parasites might be vectored by species of *Culicoides* (Ceratopogonidae) as has been shown for the monkey-infecting *Hepatocystis* species, *H. kochi* (Garnham et al. 1961). Further research is needed to explore whether the co-infections of the two different eukaryotic blood parasites are a common phenomenon in some (African) bat host species.

### Supplementary Information

Below is the link to the electronic supplementary material.

Supplementary file1 (DOCX 154 KB)

## Data Availability

All trypanosome sequences of the study are available at GenBank (NCBI) with the accession numbers PP320471–PP320496.
