# Supplementary material for: Molecular detection of trypanosomes of the Trypanosoma livingstonei species group in diverse bat species in Central Cameroon
Source: Parasitol Res. 2024 Jul 22;123(7):280. doi: 10.1007/s00436-024-08303-0 (PMC11263222; doi:10.1007/s00436-024-08303-0)
Supplement: Supplementary file 1 — Supplementary file1 (DOCX 154 KB) [file 436_2024_8303_MOESM1_ESM.docx]

**Supplementary Information**

**Molecular detection of trypanosomes of the *Trypanosoma livingstonei* species group in diverse bat species in Central Cameroon**

**Tsague KJA, Bakwo Fils EM, Atagana JP, Mbeng D, Palm L, Tchuinkam T, Schaer J**

**Supplementary Figure S1. Haplotype network analyses of trypanosomes of bats of Cameroon**

**Supplementary Table S1**. **Overview investigated bat samples and trypanosome infections**

**
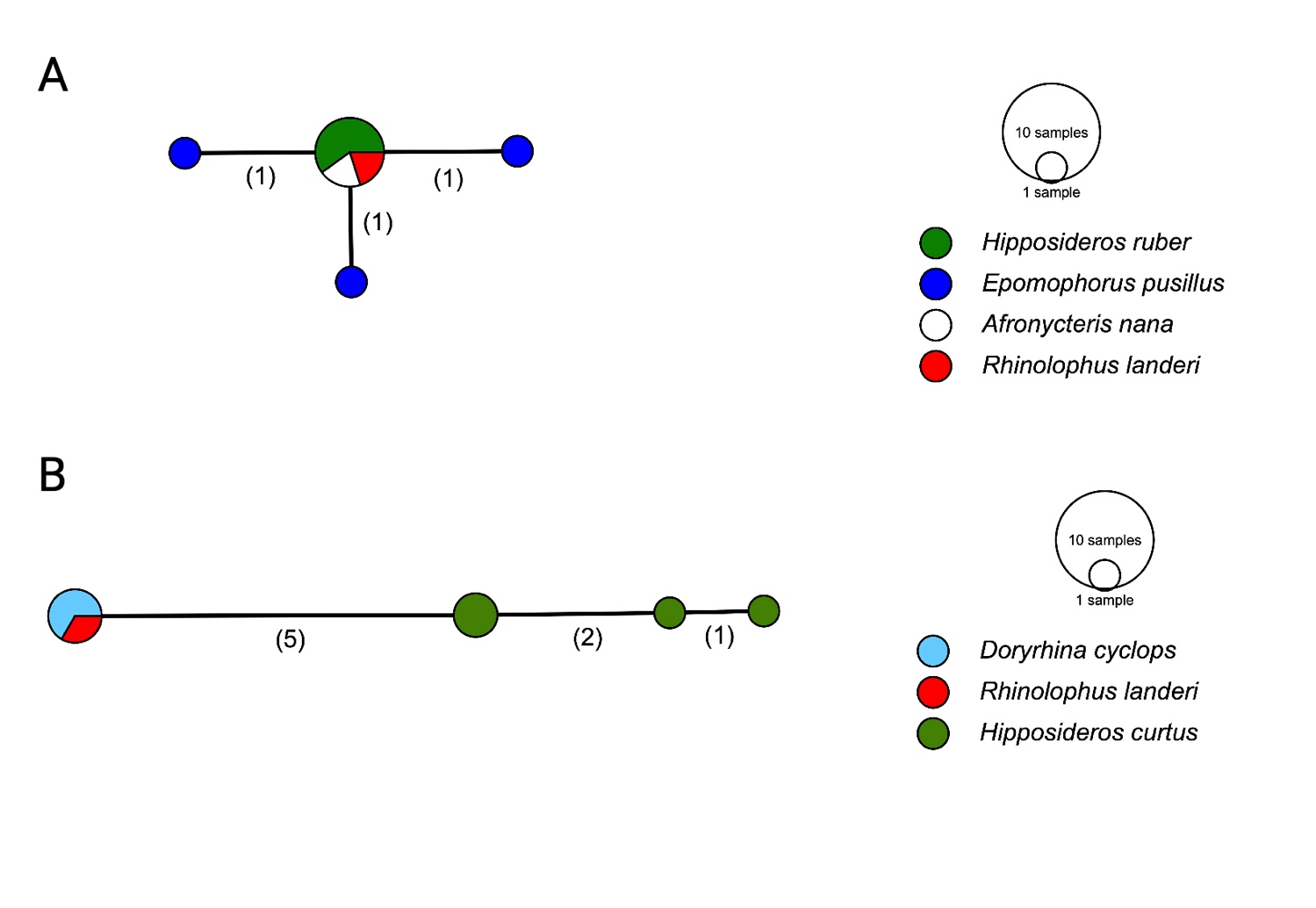
**

**Supplementary Fig. S1. Haplotype network analyses of trypanosomes of bats of Cameroon.** Median joining haplotype networks based on 18SrRNA sequences from trypanosomes detected in different bat species of Cameroon, labelled per bat species where sample was taken **(A)** Alignment of *Trypanosoma* cf. *livingstonei* sequences (n = 8; length 394 bp) **(B)** Alignment of trypanosomes (n = 7; length 451 bp) that group close to *Trypanosoma* sp. A parasites in the phylogenetic analysis. Numbers in parentheses give the number of base changes between the haplotypes.

**Supplementary Table S1**. **Overview investigated bat samples and trypanosome infections**

| **Sample #** | **Host** | ***Trypanosoma* parasite infection** | **GenBank accession number** |
| --- | --- | --- | --- |
| Nditam03 | *Afronycteris nana* | no infection |  |
| Nditam04 | *Afronycteris nana* | no infection |  |
| Nkol09 | *Afronycteris nana* | ***T.* cf. *livingstonei*** | PP320475 |
| Nkol16 | *Afronycteris nana* | no infection |  |
| Nkol19 | *Afronycteris nana* | no infection |  |
| Nditam05 | *Doryrhina cyclops* | ***Trypanosoma* sp. (co-infected with *Nycteria^a^*)** | PP320474 |
| Nditam08 | *Doryrhina cyclops* | no infection |  |
| Nditam09 | *Doryrhina cyclops* | ***Trypanosoma* sp. (co-infected with *Nycteria^a^*)** | Same haplotype as PP320474 |
| Nditam10 | *Doryrhina cyclops* | no infection |  |
| Nditam14 | *Doryrhina cyclops* | *Trypanosoma* sp.* (co-infected with *Nycteria^a^*) |  |
| Nditam15 | *Doryrhina cyclops* | no infection |  |
| Nditam16 | *Doryrhina cyclops* | *Trypanosoma* sp.* (co-infected with *Nycteria^a^*) |  |
| Nditam17 | *Doryrhina cyclops* | *Trypanosoma* sp.* (co-infected with *Nycteria^a^*) |  |
| Nditam18 | *Doryrhina cyclops* | *Trypanosoma* sp.* (co-infected with *Nycteria^a^*) |  |
| Nditam19 | *Doryrhina cyclops* | *Trypanosoma* sp.* (co-infected with *Nycteria^a^*) |  |
| Ngambé02 | *Eidolon helvum* | no infection |  |
| Ese04 | *Epomophorus pusillus* | no infection |  |
| Ese06 | *Epomophorus pusillus* | no infection |  |
| Ese10 | *Epomophorus pusillus* | no infection |  |
| Ese11 | *Epomophorus pusillus* | no infection |  |
| Ese14 | *Epomophorus pusillus* | no infection |  |
| Ese16 | *Epomophorus pusillus* | no infection |  |
| Ese18 | *Epomophorus pusillus* | no infection |  |
| Ese23 | *Epomophorus pusillus* | no infection |  |
| Ngambe01 | *Epomophorus pusillus* | no infection |  |
| Ngambe03 | *Epomophorus pusillus* | no infection |  |
| Nkol01 | *Epomophorus pusillus* | no infection |  |
| Nkol02 | *Epomophorus pusillus* | no infection |  |
| Nkol03 | *Epomophorus pusillus* | no infection |  |
| Nkol04 | *Epomophorus pusillus* | no infection |  |
| Nkol05 | *Epomophorus pusillus* | no infection |  |
| Nkol06 | *Epomophorus pusillus* | no infection |  |
| Nkol07 | *Epomophorus pusillus* | no infection |  |
| Nkol10 | *Epomophorus pusillus* | no infection |  |
| Nkol11 | *Epomophorus pusillus* | ***T.* cf. *livingstonei* (co-infected with *Hepatocystis^a^*)** | PP320471 |
| Nkol12 | *Epomophorus pusillus* | no infection |  |
| Nkol13 | *Epomophorus pusillus* | no infection |  |
| Nkol14 | *Epomophorus pusillus* | ***T.* cf. *livingstonei* (co-infected with *Hepatocystis^a^*)** | PP320472 |
| Nkol15 | *Epomophorus pusillus* | no infection |  |
| Nkol17 | *Epomophorus pusillus* | no infection |  |
| Nkol18 | *Epomophorus pusillus* | no infection |  |
| Nkol20 | *Epomophorus pusillus* | no infection |  |
| Nkol21 | *Epomophorus pusillus* | no infection |  |
| Nkol22 | *Epomophorus pusillus* | no infection |  |
| Nkol23 | *Epomophorus pusillus* | no infection |  |
| Nkol24 | *Epomophorus pusillus* | no infection |  |
| Nkol25 | *Epomophorus pusillus* | no infection |  |
| Nkol26 | *Epomophorus pusillus* | ***T.* cf. *livingstonei* (co-infected with *Hepatocystis^a^*)** | PP320473 |
| Nkol27 | *Epomophorus pusillus* | no infection |  |
| Ese01 | *Epomops franqueti* | no infection |  |
| Ese03 | *Epomops franqueti* | no infection |  |
| Ese05 | *Epomops franqueti* | no infection |  |
| Ese07 | *Epomops franqueti* | no infection |  |
| Ese08 | *Epomops franqueti* | no infection |  |
| Ese09 | *Epomops franqueti* | no infection |  |
| Ese12 | *Epomops franqueti* | no infection |  |
| Ese13 | *Epomops franqueti* | no infection |  |
| Ese15 | *Epomops franqueti* | no infection |  |
| Ese17 | *Epomops franqueti* | no infection |  |
| Ese19 | *Epomops franqueti* | no infection |  |
| Ese20 | *Epomops franqueti* | no infection |  |
| Ese21 | *Epomops franqueti* | no infection |  |
| Ese24 | *Epomops franqueti* | no infection |  |
| Nkol08 | *Epomops franqueti* | no infection |  |
| Nditam02 | *Glauconycteris humeralis* | no infection |  |
| Awoue01 | *Hipposideros abae* | no infection |  |
| Awoue02 | *Hipposideros abae* | no infection |  |
| Yoko02 | *Hipposideros curtus* | no infection |  |
| yoko100 | *Hipposideros curtus* | ***Trypanosoma* sp.** | PP320496 |
| Yoko101 | *Hipposideros curtus* | no infection |  |
| Yoko11 | *Hipposideros curtus* | no infection |  |
| Yoko12 | *Hipposideros curtus* | no infection |  |
| Yoko15 | *Hipposideros curtus* | no infection |  |
| Yoko17 | *Hipposideros curtus* | no infection |  |
| Yoko22 | *Hipposideros curtus* | no infection |  |
| Yoko26 | *Hipposideros curtus* | no infection |  |
| Yoko27 | *Hipposideros curtus* | *Trypanosoma* sp.* |  |
| yoko28 | *Hipposideros curtus* | *Trypanosoma* sp. | PP320488 |
| yoko29 | *Hipposideros curtus* | *Trypanosoma* sp.**** | PP320489 |
| Yoko40 | *Hipposideros curtus* | ***Trypanosoma* sp.** | PP320490 |
| Yoko42 | *Hipposideros curtus* | no infection |  |
| Yoko44 | *Hipposideros curtus* | no infection |  |
| Yoko45 | *Hipposideros curtus* | no infection |  |
| Yoko46 | *Hipposideros curtus* | ***Trypanosoma* sp.** | PP320491 |
| Yoko47 | *Hipposideros curtus* | ***Trypanosoma* sp.** | PP320492 |
| Yoko48 | *Hipposideros curtus* | no infection |  |
| yoko50 | *Hipposideros curtus* | *Trypanosoma* sp.* |  |
| Yoko51 | *Hipposideros curtus* | no infection |  |
| yoko56 | *Hipposideros curtus* | *Trypanosoma* sp.* |  |
| Yoko57 | *Hipposideros curtus* | no infection |  |
| Yoko58 | *Hipposideros curtus* | no infection |  |
| Yoko64 | *Hipposideros curtus* | *Trypanosoma* sp. | PP320493 |
| Yoko66 | *Hipposideros curtus* | no infection |  |
| yoko68 | *Hipposideros curtus* | ***Trypanosoma* sp.** | PP320494 |
| Yoko79 | *Hipposideros curtus* | no infection |  |
| Yoko80 | *Hipposideros curtus* | no infection |  |
| Yoko81 | *Hipposideros curtus* | no infection |  |
| Yoko82 | *Hipposideros curtus* | ***Trypanosoma* sp.** | Same haplotype as PP320490 |
| Yoko92 | *Hipposideros curtus* | no infection |  |
| Yoko93 | *Hipposideros curtus* | no infection |  |
| yoko96 | *Hipposideros curtus* | ***Trypanosoma* sp.** | PP320495 |
| Akobi35 | *Hipposideros fuliginosus* | no infection |  |
| Ngambé04 | *Hipposideros fuliginosus* | no infection |  |
| Ngambé05 | *Hipposideros fuliginosus* | no infection |  |
| Ngambé08 | *Hipposideros fuliginosus* | no infection |  |
| Ngambé12 | *Hipposideros fuliginosus* | no infection |  |
| Ngambé13 | *Hipposideros fuliginosus* | no infection |  |
| Ngambé14 | *Hipposideros fuliginosus* | no infection |  |
| Ngambé16 | *Hipposideros fuliginosus* | no infection |  |
| Ngambé19 | *Hipposideros fuliginosus* | no infection |  |
| Ngambé20 | *Hipposideros fuliginosus* | no infection |  |
| Ngambé21 | *Hipposideros fuliginosus* | no infection |  |
| Ngambé22 | *Hipposideros fuliginosus* | no infection |  |
| Ngambé23 | *Hipposideros fuliginosus* | no infection |  |
| Ngambé24 | *Hipposideros fuliginosus* | no infection |  |
| Ngambé35 | *Hipposideros fuliginosus* | no infection |  |
| Ngambé36 | *Hipposideros fuliginosus* | no infection |  |
| Ngambé38 | *Hipposideros fuliginosus* | no infection |  |
| Ngambé39 | *Hipposideros fuliginosus* | no infection |  |
| Ngambé47 | *Hipposideros fuliginosus* | no infection |  |
| Akobi14 | *Hipposideros ruber* | no infection |  |
| Akobi20 | *Hipposideros ruber* | no infection |  |
| Ngambé06 | *Hipposideros ruber* | no infection |  |
| Ngambe07 | *Hipposideros ruber* | ***T.* cf. *livingstonei*** | PP320481 |
| Ngambe30 | *Hipposideros ruber* | ***T.* cf. *livingstonei*** | Same haplotype as PP320481 |
| Ngambe32 | *Hipposideros ruber* | ***T.* cf. *livingstonei*** | Same haplotype as PP320481 |
| Nkoujou02 | *Hipposideros ruber* | no infection |  |
| Nkoujou04 | *Hipposideros ruber* | *Trypanosoma* sp.* |  |
| Nkoujou05 | *Hipposideros ruber* | *Trypanosoma* sp.* |  |
| Nkoujou07 | *Hipposideros ruber* | ***T.* cf. *livingstonei*** | Same haplotype as PP320481 |
| Nkoujou08 | *Hipposideros ruber* | *Trypanosoma* sp. | PP320479 |
| Nkoujou12 | *Hipposideros ruber* | no infection |  |
| Nkoujou16 | *Hipposideros ruber* | ***Trypanosoma* sp.** | PP320480 |
| Nkoujou21 | *Hipposideros ruber* | *Trypanosoma* sp.* |  |
| Nkoujou23 | *Hipposideros ruber* | ***Trypanosoma* sp.** | PP320482 |
| Nkoujou25 | *Hipposideros ruber* | no infection |  |
| Nkoujou28 | *Hipposideros ruber* | ***Trypanosoma* sp.** | PP320483 |
| Nkoujou29 | *Hipposideros ruber* | no infection |  |
| Nkoujou31 | *Hipposideros ruber* | ***Trypanosoma* sp.** | PP320484 |
| Nkoujou32 | *Hipposideros ruber* | ***T.* cf. *livingstonei*** | Same haplotype as PP320481 |
| Nkoujou33 | *Hipposideros ruber* | ***Trypanosoma* sp.** | PP320485 |
| Nkoujou48 | *Hipposideros ruber* | ***Trypanosoma* sp.** | PP320486 |
| Nkoujou54 | *Hipposideros ruber* | *Trypanosoma* sp.* |  |
| Nkoujou55 | *Hipposideros ruber* | ***Trypanosoma* sp.** | PP320487 |
| Yoko76 | *Hipposideros ruber* | no infection |  |
| Ngambé17 | *Rhinolophus alcyone* | no infection |  |
| Ngambé18 | *Rhinolophus alcyone* | no infection |  |
| Nditam00 | *Rhinolophus cf. landeri* | no infection |  |
| Nditam01 | *Rhinolophus cf. landeri* | no infection |  |
| Nditam06 | *Rhinolophus cf. landeri* | ***Trypanosoma* sp.** | PP320477 |
| Nditam07 | *Rhinolophus cf. landeri* | no infection |  |
| Nditam13 | *Rhinolophus cf. landeri* | ***T.* cf. *livingstonei*** | PP320478 |
| Yoko06 | *Rhinolophus cf. landeri* | ***T.* cf. *livingstonei*** | PP320476 |
| Nditam12 | *Rhinolophus landeri* | no infection |  |
| Yoko07 | *Rhinolophus landeri* | no infection |  |
| Yoko08 | *Rhinolophus landeri* | no infection |  |
| Yoko09 | *Rhinolophus landeri* | no infection |  |
| Yoko10 | *Rhinolophus landeri* | no infection |  |
| Akobi05 | *Rousettus aegyptiacus* | no infection |  |

* poor nucleotide sequence quality, but BLASTn search verified trypanosome infection

**several ambiguities in (high quality) sequence that point to mixed haplotype infection

^a^ haemosporidian parasite infection got detected and investigated in Tsague et al., 2021

**Reference**

Tsague KJA, Bakwo Fils EM, Atagana JP et al (2022) *Hepatocystis* and *Nycteria* (Haemosporida) parasite infections of bats in the Central Region of Cameroon. Parasitology 149:51-58. doi:10.1017/S0031182021001542
